# Comparative Analysis of the Postoperative Analgesic Effects of Caudal Epidural Injection of Ropivacaine Combined With Fentanyl Versus Ropivacaine Alone in Lumbosacral Spine Surgeries: A Randomized Double-Blinded Study

**DOI:** 10.7759/cureus.70963

**Published:** 2024-10-06

**Authors:** Suresh Rajwade, Rashmi Dubey, Monica Khetarpal, Sarita Ramchandani, Chinmaya K Panda, Mayank Kumar, Jitendra V Kalbande

**Affiliations:** 1 Anaesthesiology, All India Institute of Medical Sciences, Raipur, Raipur, IND; 2 Anesthesiology and Critical Care, All India Institute of Medical Sciences, Raipur, Raipur, IND

**Keywords:** caudal epidural block, fentanyl, lumbosacral spine surgeries, postoperative analgesia, ropivacaine

## Abstract

Introduction: A single injection of local anesthetic as a caudal epidural block provides pain relief for 2-4 hours. This duration can be extended by adding adjuvants such as opioids (morphine, fentanyl, buprenorphine, tramadol), ketamine, α2 agonists (dexmedetomidine, clonidine), and adrenaline. Caudal analgesia also reduces the need for intravenous opioids during and after surgery, which helps avoid the systemic side effects of opioids. Additionally, adjuncts such as opioids synergistically augment the analgesic properties of caudal epidural anesthetics without escalating motor block. Combining local anesthetics and opioids also reduces the dose-related adverse effects of each drug.

Materials and method: Fifty-six ASA (American Society of Anesthesiologists) grade I and II patients undergoing lumbosacral spine surgeries were randomized into two groups. The RF group (n=28) received a single caudal epidural injection of 20 ml of 0.2% ropivacaine with 50 micrograms of fentanyl, while the R group (n=28) received 20 ml of 0.2% ropivacaine alone. Postoperatively, patients were monitored for pain levels, heart rate (HR), non-invasive blood pressure (NIBP), mean arterial pressure (MAP), and visual analog scale (VAS) scores. The time until the first rescue analgesia request and the total amount of rescue analgesia administered were also recorded.

Results: The two groups were comparable in terms of age, weight, height, and body mass index (BMI). In the R group, the VAS score was at least 1.82 at 2 hours and at most 5.96 at 6 hours, then decreased to 2.25 at 24 hours. In the RF group, the VAS score was at least 1.68 at 2 hours and at most 5.87 at 4 hours, then decreased to 2.29 at 24 hours. In the RF group, the time until the first rescue pain relief was needed was significantly longer compared to the R group (in RF, the mean value was 7.30 hours, and in R, 6.68 hours, p <.0001).

Conclusions: The study shows that adding 50 micrograms of fentanyl to 20 ml of 0.2% ropivacaine for ultrasound-guided caudal block in patients undergoing lumbosacral spine surgeries results in longer analgesia duration and reduced VAS scores over the postoperative 24 hours.

## Introduction

Caudal anesthesia was first described by Fernand Cathelin and Jean-Anthanase Sicard in the last century. However, it did not gain popularity due to wide variations in sacral bone anatomy and a failure rate of 5% to 10%. In the 1940s, there was a renewed interest in caudal anesthesia led by Hingson and colleagues, who used it in obstetric anesthesia. Caudal epidural block is now a widely utilized medical procedure and serves as an effective method for providing surgical anesthesia in both pediatric and adult patients. Additionally, it is instrumental in the treatment and management of both acute and chronic pain conditions. It can achieve 98%-100% success rates in infants and young children before puberty and lean adults [1]. Caudal injection given via sacral hiatus is easy to perform due to the injection site being distal from the surgical site. Also, it does not increase the risk of infections or CSF (cerebrospinal fluid) leakage [2]. Preemptive caudal analgesia can effectively be used in lumbosacral spine surgery. The conventional method to relieve pain in lumbosacral spine surgery is the parental injection of analgesics like opioids and nonsteroidal anti-inflammatory drugs (NSAIDS) [3]. After injection of local anesthetic agents in the epidural space, neural blockade and fixation of the drug occur in about 20 minutes. A single injection of local anesthetic as caudal epidural block provides analgesia for 2-4 hours; however, this can be prolonged by adding adjuvants such as opioids (morphine, fentanyl, buprenorphine, tramadol), ketamine, α2 agonists (dexmedetomidine, clonidine), and adrenaline [4]. The overall advantage of intravenous analgesia over caudal analgesia is still controversial, although many studies have shown that caudal epidural provides superior postoperative analgesia as compared to intravenous analgesia [5-7]. Another notable benefit of caudal analgesia is that it reduces intraoperative and postoperative intravenous opioid requirements, thus avoiding systemic side effects of opioids. Adjuvant-like opioids synergistically increase the analgesic effects of caudal epidural anesthetics without increasing motor block. Independently Combining local anesthetics and opioids decreases each drug's dose-related adverse effects. The epidural opioids, like fentanyl, because of their lipophilic nature, readily diffuse across the dura and arachnoid matter and reach the CSF and dorsal horn of the spinal cord. Afterward, it is absorbed by the epidural vasculature and reaches systemic circulation for metabolism and excretion.

Several studies suggest that it is the principal analgesic mechanism. The onset of action of epidural fentanyl is 5 to 15 minutes and can be given as a bolus dose of 10 to 100 micrograms to provide analgesia [8].

In our study, we aimed to compare the quality and duration of pain relief using a combination of caudal epidural ropivacaine and fentanyl versus using ropivacaine alone in lumbosacral spine surgeries. We hypothesized that adding fentanyl to ropivacaine injections increases the duration and quality of postoperative analgesia for patients undergoing lumbosacral spine surgeries.

## Materials and methods

This single-center prospective randomized double-blinded study was carried out at the operation theatre and postoperative wards in AIIMS, Raipur, after approval from IEC AIIMS Raipur. IEC proposal number: AIIMSRPR/IEC/2020/681. CTRI registration no. is CTRI/2022/01/039415.

The aim of this study was to assess and compare the analgesic effects of caudal epidural injection of ropivacaine alone versus ropivacaine combined with fentanyl in the postoperative setting. The primary outcome of the study was to assess the quality of analgesia by measuring the reduction in the requirement of rescue analgesia and VAS score postoperatively. The secondary outcomes included assessing the duration of analgesia (the time to first rescue analgesia), the 24-hour VAS score, and any adverse effects.

Patients aged 18 to 65 years, of both sexes and ASA grades 1 and 2, who were scheduled for elective lumbosacral spine surgery under general anesthesia and provided written informed consent, were included in the study. Patients with uncontrolled hypertension, diabetes mellitus, and asthma, and who were prescribed opioids in any form preoperatively were excluded. Patients with a history of lumbosacral surgery, allergy to ropivacaine or fentanyl, and lumbosacral anomalies were also excluded from the study.

The sample size was calculated based on a previously published study (Shashwat Kumar et al., 2016) [3].

Formula 

$n=(Z_(1-&alpha;/2)^(2 \sigma^2 ))/d^2$​

Where σ =Standard deviation, d= Precision, 1-α/2= Desired confidence level

Substituting the values into the formula, the estimated sample size was 25 for each group. Since we had two groups, the total sample size was 50. To optimize our results, we ultimately chose to include 56 patients, with 28 in each group. 

For randomization and blinding, we used stratified randomization to control and balance the influence of covariates (Table 1).

**Table 1:** Stratified randomization

Male		Female	
Age (18-40)	Age (41-65)	Age (18-40)	Age (41-65)
14	14	14	14

For each group, a random selection of patients was made using the lottery method, and they were grouped according to their study group.

Group allocation and allocation concealment 

Group A: Ropivacaine 20 ml at 0.2% concentration with fentanyl 50mcg. Group B: Ropivacaine 20 ml at 0.2% concentration.

Group allocation was concealed in a sealed, opaque envelope, randomly picked up by the patient in the preoperative area. The envelope was opened up by a qualified healthcare worker (HCW) who was not part of the study. The HCW then prepared the study drugs as per the group allocation and handed them over to the anesthesiologist performing the procedure, and the person collecting data and providing postoperative care was blinded to group allocation.

Method

A preoperatively detailed history, clinical examination, and pre-anesthetic checkup (PAC) were done. The entire anesthetic procedure, including the drugs, was explained to the patients. Informed written consent was taken. Investigations were carried out as per institutional protocol. The visual analog score (VAS) for pain scoring was explained to patients. The VAS has endpoints (0 to 10) labeled zero as no pain and ten as worst pain. Patients were kept nil per oral as per ASA standards for solids, liquids, and clear fluids. In the operative room, standard ASA (American Society of Anesthesiologists) monitors were attached to the patient (electrocardiogram, non-invasive blood pressure, and pulse oximeter), and baseline parameters were recorded. Intravenous access was established with an 18-G intravenous cannula under local anesthesia. Anesthesia was induced with propofol (2mg/kg), injection of fentanyl (2 microgram/kg and vecuronium bromide (0.1 mg/kg) was administered to facilitate endotracheal intubation. After securing the airway, the patient's lung was mechanically ventilated using nitrous oxide in oxygen (50%:50%) with an end-tidal CO2 between 30 and 35 mm Hg. Anesthesia was maintained with sevoflurane (MAC 1-1.2). Injection of Vecuronium in supplemental doses was given after assessment of neuromuscular function with a train of four. Rescue analgesia in the form of a bolus of 0.5 micrograms/kg of fentanyl was given when more than a 20% rise in heart rate or blood pressure was observed. After intubation, the patient was turned into a prone position for surgery. Ultrasound-guided caudal epidural block with drugs as per group allocation was given 20 minutes prior to the surgical incision. Group A: Received 20 ml of ropivacaine at 0.2% concentration with fentanyl 50 mcg. Group B: Received 20 ml of ropivacaine at 0.2% concentration. After completion of the surgery, the patient was reversed and extubated as per protocol. Hemodynamic monitoring was continuous throughout the surgery, and adverse events were recorded and treated. Total rescue doses of fentanyl were noted and recorded. Postoperatively, heart rate (HR), noninvasive blood pressure (NIBP), mean arterial blood pressure (MAP), and oxygen saturation (SPO2) were monitored and noted every second hour for the first 12 hours and fourth hourly for the next 12 hours. Any episodes of hypotension (20% decrease in mean arterial pressure in relation to baseline values), bradycardia (HR <50 beats/min), or hypoxemia (spo2 <90%) were recorded and treated. Postoperatively, intravenous (IV) paracetamol (1gm) was given eighth hourly in both groups. The pain was assessed using a visual analog score (VAS) every 2nd hour for the first 12 hours and then four hourly for the next 12 hours. The VAS score was described as 0-no pain, 1 to 3 mild pain, 4 to 7 moderate pain, and 8 to 10 severe pain. Injection Fentanyl 1mcg/kg was administered when VAS >3. The time of the first rescue analgesic demand was recorded, and the duration of analgesia was considered to be due to the caudal block. The patient was reassessed after 1 hour. In case VAS was still >3, an injection of diclofenac 50 mg was given as rescue analgesia or an injection of tramadol 50 mg where diclofenac was contraindicated. A total dose of rescue analgesia was calculated and recorded.

Data analysis

Statistical analysis was carried out using statistical packages for SPSS Inc. Released 2007. SPSS for Windows, Version 16.0. Chicago, SPSS Inc. Continuous and categorical variables were expressed as mean ± SD and percentages. The Wilcoxon-Mann-Whitney U test was applied to compare the two groups. The Friedman test was used to compare at different time intervals. Categorical variables were compared using the chi-square test. Two-sided p values were considered statistically significant at p<0.05.

## Results

The present study included 56 ASA I-II patients undergoing lumbosacral spine surgeries under general anesthesia. Patients were randomized into two groups, R and RF. There was no statistical difference between the two groups with respect to age, gender, weight, height, and BMI. This study included patients of ASA grades 1 and 2 only. 85.7% of patients are of ASA grade 1, and 14.3% of patients are of ASA grade 2.

Intraoperative hemodynamics: There was no statistically significant difference between the two groups in respect of intraoperative HR and MAP. Patients of both groups were stable hemodynamically throughout the intraoperative period. 

The intraoperative requirement of fentanyl was 2.86 ± 10.84 micrograms in group R and 3.57±18.90 micrograms in group RF. There was no statistically significant difference between the groups regarding Total Intra-Operative Fentanyl Dose (W = 405.000, p = 0.600) (Table 2).

**Table 2:** Total intraoperative fentanyl requirement

Total Intra-Operative Fentanyl Dose (in micrograms)	Group		Wilcoxon-Mann-Whitney U Test	
	R	RF	W	p value
Mean (SD)	2.86 (10.84)	3.57 (18.90)	405.000	0.600
Median (IQR)	0 (0-0)	0 (0-0)		
Min - Max	0 - 50	0 - 100		

After administration of caudal epidural block, only three patients required intraoperative supplementation with injection of fentanyl IV. In group RF, only one patient required an analgesic supplement and was given 100 micrograms of injection fentanyl IV. In group R, only two patients required analgesic supplements; one patient required 30 micrograms, and another patient required 50 micrograms of injection fentanyl IV.

Postoperatively, the mean duration of analgesia was 6.68 (0.54) hours in group R and 7.30 (0.42) hours in group RF. Between the two groups, there was no significant difference in the use of postoperative analgesia (t = -4.766, p = <0.001), with the mean postoperative analgesia being highest in the RF group. Strength of Association (Point-Biserial Correlation) = 0.54 (Large Effect Size) (Table 3).

**Table 3:** Time to first rescue analgesia (duration of postoperative analgesia)

Duration of Post-Operative Analgesia (in hours)	Group		t-test	
	R	RF	t	p-value
Mean (SD)	6.68 (0.54)	7.30 (0.42)	-4.766	<0.001
Median (IQR)	6.79 (6.25-7.08)	7.42 (7.13-7.58)		
Min - Max	5.75 - 7.75	6.42 - 7.92		

The two groups differed significantly in terms of VAS at the following time points: 4 hours and 10 hours.

In Group R, the mean VAS increased from a minimum of 1.82 at the 2-hour timepoint to a maximum of 5.96 at the 6-hour timepoint and then decreased to 2.25 at the 24-hour timepoint. This change was statistically significant (Friedman Test: χ2 = 80.6, p = <0.001).

In Group: RF, the mean VAS increased from a minimum of 1.68 at the 2-hour timepoint to a maximum of 5.86 at the 4-hour timepoint and then decreased to 2.29 at the 24 hour timepoint. This change was statistically significant (Friedman Test: χ2 = 90.1, p = <0.001) (Table 4).

**Table 4:** Comparison of the two groups in terms of change in postoperative VAS over time

VAS	Group		p-value for comparison of the two groups at each of the timepoints (Wilcoxon-Mann-Whitney Test)
	R	RF	
	Mean (SD)	Mean (SD)	
2 Hours	1.82 (1.39)	1.68 (1.06)	0.584
4 Hours	5.14 (1.43)	5.86 (1.11)	0.027
6 Hours	5.96 (1.67)	5.25 (1.80)	0.057
8 Hours	2.96 (1.77)	3.79 (2.04)	0.111
10 Hours	3.21 (1.26)	2.64 (1.31)	0.009
12 Hours	3.36 (1.99)	3.07 (1.76)	0.587
16 Hours	5.07 (1.92)	5.00 (2.45)	0.861
20 Hours	4.07 (2.54)	3.25 (2.07)	0.332
24 Hours	2.25 (1.67)	2.29 (0.90)	0.744
p-value for change in VAS over time within each group (Friedman Test)	<0.001	<0.001	
Overall p-value for comparison of change in VAS over time between the two groups (Generalized Estimating Equations)	0.003		

In both groups, after the second hour of the postoperative period, the VAS score was less than four, so rescue analgesia was not given. 

As postoperative rescue analgesia, fentanyl was administered when the VAS exceeded 3. Due to its short duration of action, it was supplemented with diclofenac. For patients contraindicated for diclofenac, tramadol was used instead. The requirement of injection fentanyl as rescue analgesia was 123.21 micrograms in the R group and 126.79 micrograms in the RF group (p-value 0.765) (Table 5). No adverse effects or complications related to the block or drugs were observed.

**Table 5:** Comparison of the two groups as total postoperative rescue analgesia

Total postoperative rescue analgesia	Group		Wilcoxon-Mann-Whitney U Test	
	R	RF	W	p-value
Fentanyl Mean (SD)	123.21 (44.06)	126.79 (37.22)	374.500	0.765
Diclofenac Mean (SD)	64.29 (40.50)	64.29 (40.50)	392.000	1.000
Tramadol Mean (SD)	14.29 (29.99)	7.14 (17.82)	424.000	0.438

## Discussion

In this study, 56 patients of ASA grade I-II undergoing lumbosacral spine surgeries under general anesthesia were included. The patients were randomly divided into two groups: Group R, which received 20 mL of 0.2% Ropivacaine, and Group RF, which received 20 mL of 0.2% Ropivacaine along with 50 micrograms of fentanyl in a caudal epidural block. There were no significant differences between the two groups in terms of age, weight, height, and BMI. Only three patients required intraoperative fentanyl injection. The average intraoperative fentanyl consumption was 2.86 micrograms in Group R and 3.57 micrograms in Group RF. The patients in the R group showed higher VAS scores than the RF group. In the R group, the minimum VAS score was 1.82 at 2 hours, and the maximum VAS score was 5.96 at 6 hours. The VAS score decreased to 2.25 at 24 hours. In the RF group, the minimum VAS score was 1.68 at 2 hours, and the maximum VAS score was 5.87 at 4 hours. The VAS score then decreased to 2.29 at 24 hours. In the RF group, the duration for the first rescue analgesia requirement was significantly higher than in the R group. The mean duration for first rescue analgesia in the RF group was 7.30 hours and in the R group was 6.68 hours (p <.0001). The requirements of rescue analgesia in postoperative 24 hours in both groups were not statistically significant. In the RF group injection of fentanyl required postoperatively was 126.79 micrograms, and in the R group 123.21 micrograms (p value). Injection Diclofenac required postoperatively was 64.2 mg in RF and 64.2 mg in R, and Injection Tramadol was 14.29 mg in RF and 7.14 mg in R group.

The caudal epidural block was effective in all patients, and no adverse effects or complications related to the block or drugs were observed. Adding fentanyl to ropivacaine in the caudal epidural block resulted in lower postoperative VAS scores and a longer duration of pain relief compared to using ropivacaine alone.

Patients undergoing surgical laminectomy frequently experience severe postoperative pain, which can hinder mobilization and physiotherapy, prolonging hospital stays and recovery times [9,10].Poorly controlled pain also increases the risk of complications such as deep venous thrombosis, pulmonary embolism, and infections [11]. Effective pain management is essential to improve patient outcomes and expedite recovery.

Caudal epidural anesthesia primarily works by blocking the spinal roots, offering a quick and efficient method for surgical anesthesia and postoperative pain relief with a lower cost compared to interlaminar epidural blocks. It generally poses minimal risk of neurological deficits if done correctly, though there can be a 10% rate of technical failure due to anatomical variations, such as an absent sacral hiatus [12]. For our procedures, we used ropivacaine 0.2% and fentanyl for its safety benefits. Ropivacaine is an aminoamide, long-acting LA agent. Ropivacaine shows apparent sensory motor separation, producing sensory nerve Aδ and c fiber blockage while leaving the motor function of α fibers largely unaffected [13]. Ropivacaine has a better safety profile than other local anesthetics regarding CNS and cardiac toxicity. Epidural and intrathecal administration of fentanyl is a long-established route for intraoperative anesthesia and postoperative analgesia. It is also associated with fewer adverse cardiovascular effects than morphine and triggers substantially less histamine release. The lipophilic properties of fentanyl were thought to confer minimal risk of delayed respiratory depression due to poor cephalad spread in the CSF [14]. 

Shashwat Kumar et al. conducted a study that shows that pre-emptive caudal ropivacaine provides effective analgesia during degenerative lumber spine surgery. In this study, they compared 0.2% caudal Ropivacaine 20 mL with intravenous analgesia and found that the average time interval of first rescue analgesia was 8.47 hours in the study group and 1.10 hours in the control group [3]. In our study, we found the average time for first rescue analgesia with ropivacaine was 6.68 hours, and ropivacaine with fentanyl was 7.30 hours. Shashwat Kumar et al. found that the total intraoperative fentanyl required was higher (average 171 µg) in the control group than in the study group (average 143 µg), but this difference was not statistically significant [3]. In our study, we found a significantly lower requirement of intraoperative fentanyl in both groups.

Sekar et al. conducted a study that showed that pre-emptive caudal Ropivacaine is effective for analgesia during lumbosacral spine surgery. In this study, they compared 0.375% Bupivacaine and 50 mg Tramadol with an injection of 20 ml of normal saline in the control group. They found that the mean time interval at which the first demand for rescue analgesia was significantly higher than the control group [15].

Nagappa et al. conducted a study on Clonidine as an adjuvant to caudal epidural Ropivacaine for lumbosacral spine surgery. They found that the addition of Clonidine 1 microgram/kg in Ropivacaine 0.2% gave better postoperative analgesia than Ropivacaine alone. The RC group required a longer time for the first rescue analgesia than the R group, with a mean ± SD of 3.10 ± 3.23 and 2.97 ± 4.86, which was statistically significant (p = 0.011) [16].

Kalappa et al. conducted a study on dexmedetomidine used as an adjuvant to pre-emptive caudal epidural Ropivacaine for lumbosacral spine surgery. They randomized the patients into groups: Ropivacaine 0.2% (R group) or a mixture of Ropivacaine 0.2% and Dexmedetomidine 1 microgram/kg (RD group). They found mean VAS scores were significantly lower in the RD group for up to 12 hours following the caudal block. The result suggests that injection dexmedetomidine is an effective additive to injection ropivacaine as preemptive analgesia when given by caudal epidural route in patients posted for lumbosacral spine surgeries. Time to rescue analgesia in Group RD ranged from 420-444 min [Avg=432(7.2hours), SD=6.70], and in a group, R was 422-490 min (Avg=456, (7.6 hours) SD=10.89) [17]. In our study, the time to first rescue analgesia in the R group is 6.68 and RF 7.30, almost similar to this study group.

Tarlika P. Doctor et al. conducted a study on a comparison of Ropivacaine and Bupivacaine with Fentanyl for a caudal epidural in pediatric surgery. All the patients were randomly divided into groups RF (Ropivacaine 0.2% 2mg/kg + Fentanyl 1 microgram/kg) and BF (Bupivacaine 0.25% 2mg/kg + Fentanyl 1 microgram/kg). The duration of analgesia was prolonged in both groups, RF and BF. The time for the first rescue analgesic requirement for group RF was 6.1 ± 1.1 hours, and for group BF, it was 5.6 ± 0.9 hours. They found that ropivacaine with fentanyl is a better combination for pediatric surgeries as an adjuvant to general anesthesia [18].

Our study demonstrates that administering preemptive caudal epidural injection of ropivacaine plus fentanyl results in increased duration and improved quality of pain relief, as well as a reduction in the consumption of intraoperative injection fentanyl. However, postoperative consumption of fentanyl, paracetamol, and tramadol was found to be almost similar in both groups.

Limitations of the study

The study encounters some limitations that may impact its findings. The absence of a control group receiving a placebo or an alternative standard treatment limits the ability to fully evaluate the relative effectiveness of ropivacaine with fentanyl. Additionally, the subjective nature of the Visual Analog Scale (VAS) for pain, coupled with variability in individual pain perception and the timing of assessments, may affect the accuracy of the results. External factors, such as concurrent medications or differing pain management practices, could also influence the outcomes and complicate comparisons.

## Conclusions

The study found that administering a caudal epidural injection of 0.2% ropivacaine alone or in combination with 50 micrograms of fentanyl resulted in improved postoperative pain relief for patients undergoing lumbosacral spine surgeries. The use of caudal epidural injection also led to decreased intraoperative and postoperative opioid consumption. Adding 50 micrograms of fentanyl to 20 ml of 0.2% ropivacaine for ultrasound-guided caudal epidural block in patients undergoing lumbosacral spine surgeries ensures better quality, longer duration of analgesia, and lower VAS scores over the postoperative 24 hours.
